# Effects of berberine hydrochloride on antioxidant response and gut microflora in the *Charybdis japonica* infected with *Aeromonas hydrophila*

**DOI:** 10.1186/s12866-024-03420-3

**Published:** 2024-08-02

**Authors:** Mingming Han, Yanxia Guo, ShengKai Tang, Daming Li, Jinjuan Wan, Chenxi Zhu, Zakaria Zuraini, Ji Liang, Tianheng Gao, Zihan Zhou, Qichen Jiang

**Affiliations:** 1 Freshwater Fisheries Research Institute of Jiangsu, 79 Chating East Street, 210017 Nanjing, China; 2 Biology Program, School of Distance Education, Universiti Sains Malaysia, 11800 Minden, Penang Malaysia; 3 Centre for Marine and Coastal Studies, University Sains Malaysia, Minden, 11800 Malaysia; 4 Institute of Marine Biology, College of Oceanography, Hohai University, 210024 Nanjing, China; 5 College of Animal Science and Technology, Sichuan Agricultural University, Chengdu, 611130 Sichuan China

**Keywords:** Berberine hydrochloride, *Charybdis japonica*, 16S rRNA, Intestinal microbes, *Aeromonas hydrophila*

## Abstract

This study used berberine hydrochloride to treat the Asian paddle crab, *Charybdis japonica* infected with the Gram-negative bacterium *Aeromonas hydrophila* at concentrations of 0, 100, 200 and 300 mg/L. The effect of berberine hydrochloride on the survival rate and gut microbiota of *C. japonica* was investigated. Berberine hydrochloride improved the stability of the intestinal flora, with an increase in the abundance of probiotic species and a decrease in the abundance of both pathogenic bacteria after treatment with high concentrations of berberine hydrochloride. Berberine hydrochloride altered peroxidase activity (POD), malondialdehyde (MDA), and lipid peroxidation (LPO) in the intestinal tract compared to the control. Berberine hydrochloride could modulate the energy released from the enzyme activities of hexokinase (HK), phosphofructokinase (PFK), and pyruvate kinase (PK) in the intestinal tract of *C. japonica* infected with *A. hydrophila.* Zona occludens 1 (ZO-1), Zinc finger E-box binding homeobox 1 (ZEB1), occludin and signal transducer, and activator of transcription5b (STAT5b) expression were also increased, which improved intestinal barrier function. The results of this study provide new insights into the role of berberine hydrochloride in intestinal immune mechanisms and oxidative stress in crustaceans.

**Supplementary Information:**

The online version contains supplementary material available at 10.1186/s12866-024-03420-3.

## Introduction

Bacterial infection is the main cause and limiting factor of disease in crab cultures [1–3]. *Aeromonas hydrophila* infects through the occasional abrasions of fish and crabs and the removal of phosphorus sheets [4, 5]. In recent years, *A. hydrophila* has infected many fish, including *Ctenopharyngodon idella* [6], *Cyprinus carpio* [7] and *Megalobrama amblycephala* [8]. *A. hydrophila* is the main causal agent of sepsis [9], and the external symptoms of this disease are hemorrhagic spots [10].

Microbiota and its products are essential for shaping the development and function of the host's innate immune system, thus influencing intestinal health [11]. Therefore, the intestinal flora play an important physiological and functional role in crabs. Intestinal flora can affect the normal structure and functional development of the mucosal immune system and have the ability to transform endogenous and dietary substrates into absorbable nutrients and resist the invasion of external bacteria [12]. It has been widely established that the intestinal flora is highly host-related. It is involved in metabolism, synthesis of many important biochemical substances, and the regulation of gene expression, and plays a role in the development of host tissues and organs, and the maturation of the immune system [2]. High-throughput 16S rDNA sequencing studies of crab intestinal flora diversity have been undertaken to some scale. The composition of the intestinal microbial community of different crabs has been described and characterized, such as *Eriocheir sinensis* [13], *Scylla paramamosain* [14], and *Portunus pelagicus* [15]. By comparing the intestinal microflora of healthy and diseased crabs, it was found that the relative abundance of bacteria in healthy crabs was 3–4 times higher than in diseased crabs [16]. *Tenericutes, Epsilonproteobacteria*, and *Gammaproteobacteria* were dominant in the intestinal flora of cultured animals [17]. The composition of the intestinal flora in *C. japonica* has been rarely reported. Therefore, the effect of pathogenic bacteria on intestinal flora in this species needs further study.

With the continuous in-depth study of natural drugs, more antibacterial and bactericidal active ingredients have been found, including alkaloids [18]. The main alkaloid of *Coptis chinensis* is berberine hydrochloride, an isoquinoline alkaloid [19]. It has been shown that berberine hydrochloride has a strong inhibitory effect on *Staphylococcus aureus *in vitro and has a good inhibitory effect on a variety of pathogenic fungi isolated from the human body, such as *Candida albicans* [20]. Berberine hydrochloride has bacteriostatic and bactericidal effects on *Vibrio cholerae* and *Escherichia coli* [21]. By reducing the activity of acetyltransferase, berberine hydrochloride causes metabolic disorders of arylamines in *Salmonella typhimurium*, leading to the death of the bacteria due to the failure of normal metabolism [22]. Berberine hydrochloride not only has hypoglycemic and microbial pharmacological effects, but also has other extensive pharmacological effects, such as antiplatelet agglutination [23]. Berberine hydrochloride and its derivatives can combine with single- or double-stranded DNA to form complexes and inhibit bacteria [24]. It can also reduce the synthesis of bacterial DNA by inhibiting the activity of topoisomerase 1 and topoisomerase 2 and can prevent further inflammation by inhibiting neutrophil infiltration [25].

In this study, *C. japonica* was treated with berberine hydrochloride and then injected with *A. hydrophila*. The antimicrobial pharmacological effects of berberine hydrochloride on the composition of the intestinal flora of *C. japonica* were assessed to preliminarily explore the relationship between structural changes of intestinal flora and the sensitivity of pathogenic bacteria. The effects of berberine hydrochloride on the intestinal barrier function, oxidative stress and glucose metabolism of *C. japonica* were assessed.

## Materials and methods

### Organization and materials

To check the protective effects of berberine hydrochloride, two experiments were designed. One experiment studied survival rates under *A. hydrophila* under challenge, while the second experiment investigated the mechanism of action of berberine hydrochloride. *Charybdis japonica,* which was obtained from the sea of Malaysia, weighed 80 ± 2.4 g and measured 8 ± 1.5 cm in length and 6 ± 2.8 in width. PVC pipes were used as animal shelters to prevent cannibalism. During a 2-week acclimation period, all crabs were fed commercial feed (9812; Shanghai Harmony Feed Co. Ltd., China) at 7:00 and 20:00 every day. The crabs were fed a daily regimen consisting of a quantity of food equivalent to 5% of their body weight. Seawater exposure was implemented through an artificial sea salt cycle, maintaining a salinity level of 28 practical salinity units (psu). The water temperature was controlled within a range of 25 ± 1 °C, while the pH level was set at 8.0 ± 0.2. The concentration of dissolved oxygen in the water was maintained at 5.0 mg L − 1. The light/dark cycle was set to 12 h.

For making sure the protection effects of berberine hydrochloride, a total of 720 *C. japonica* were used. The *C. japonica* were equally divided into four treatment groups (3 × 30 *C. japonica*/tank), named AH, BHAH1, BHAH2, and BHAH3. *C. japonica* in each group were soaked in 0, 100, 200 and 300 mg/L berberine hydrochloride for 48 h, respectively, then injected with 10^5^ CFU/L of *A. hydrophila*. The number of surviving crabs was checked at 36, 120, and 168 h.

Based on the results of mortality rate analyses, another experiment of gut health was carried out. A total of 288 *C. japonica were* divided into 8 groups (3 × 12 *C. japonica*/tank), half groups soaked.in 0, 100, 200 and 300 mg/L berberine hydrochloride for 48 h were named CK, BH1, BH2 and BH3, respectively. another groups were soaked in 0, 100, 200 and 300 mg/L berberine hydrochloride for 48 h and then injected with 10^5^ CFU/L *A. hydrophila.* The groups were named AH, BHAH1, BHAH2, and BHAH3, respectively. Crabs were collected and anesthetized on ice after 72 h. Intestinal tissues were collected with sterile scissors and tweezers, and twelve guts were combined into one sample (6 samples were used for Illumina MiSeq sequencing, 3 samples were used for gene expression, and 3 samples were used for biochemical tests). RNA isolation was performed on ice.

### The process of extracting DNA and amplifying it through polymerase chain reaction (PCR)

The microbial DNA present in the crab's gut was isolated using the E.Z.N.A. Soil DNA Extraction Kit (Omega Bio-Tek Inc., Norcross, GA, USA.) The assessment of both DNA quantity and quality was conducted using a NanoDrop 2000 spectrophotometer (Thermo Fisher Scientific, DE, USA). Additionally, 1% agarose gel electrophoresis was employed for this purpose. The V3 V4 region of the 16S bacterial ribosomal RNA (rRNA) gene was amplified by PCR. The amplification process utilized primers 341F (5'-CCTACGGGNGGCWGCAG-3') and 805R (5'-GACTACHVGGGTATCTAATCC-3'). The PCR itself was conducted using the GeneAmp 9700 thermal cycling system, manufactured by Applied Biosystems (Foster City, CA, USA). Subsequently, the PCR products underwent separation via 2% agarose gel electrophoresis, and purified using the AxyPrep DNA gel extraction kit (Axygen, Union City, CA, USA). Quantification of the PCR products was performed using the Quantifluor-st fluorescence analyzer manufactured by Promega (Madison, WI, USA).

### Illumina Miseq sequencing

A total of 600 libraries were generated by purifying and amplifying fragments following the established protocols of the Illumina MiSeq platform (Illumina, San Diego, CA, USA). The raw sequences were then imported into FASTQ files for subsequent analysis using QIIME2. To process the data, the QIIME2 DADA2 plug-in was utilized for quality control, trimming, denoising, merging, and removal of chimeras, resulting in the creation of the final feature sequence table. The Amplicon Sequence Variants (ASVs) obtained from the representative samples were further compared to sequences in the GREENGENES database, employing a similarity threshold of 99%. For this comparison, the database was filtered to include only sequences corresponding to the primer pairs. Consequently, a classification information table of species was acquired, and any contaminating mitochondria were effectively eliminated from the dataset.

To discern variations in bacterial abundance across different groups, we employed statistical techniques such as analysis of variance (ANOVA) using the "mixOmics" software package in R. Furthermore, Partial Least Squares Discriminant Analysis (PLS-DA), a supervised statistical technique for discrimination, was utilized to construct a model that establishes the correlation between microbial communities and sample categories. By analyzing the relative abundance of prominent microbial species present in the samples, we were able to make predictions regarding sample categories. In addition, a co-occurrence analysis was conducted to calculate Spearman rank correlation coefficients, thereby offering valuable insights into the interrelationships among various species. Additionally, the PICRUSt software was employed to generate predictions regarding the potential functional composition of the microbiota. The analysis was conducted using default settings, unless otherwise specified. The data pertaining to this study have been archived in the repository of the National Center for Biotechnology Information (NCBI). The accession numbers for the data are as follows: SRA: SRP358796 and Bioproject accession: PRJNA804319.

### Trehalose, hexokinase, pyruvate kinase activity, and trehalase (THL) activity results

Intestinal samples (100 mg) homogenized in 400–800 μL frozen PBS (pH 7.4) was centrifuged at 14,000 rpm and 4 °C for 20 min. The recovered supernatant was placed on ice for testing. Trehalose, trehalase (THL), Hexokinase (HK), phosphofructokinase (PFK), pyruvate kinase (PK), peroxidase (POD), malondialdehyde (MDA), and lipid peroxidation (LPO) were determined using a commercial kit (Jiancheng Institute of Bioengineering, Nanjing, China). MDA, POD, and LPO were used to assess the ability of berberine hydrochloride to cope with oxidative stress in the intestine. The activities of HK, THL, PFK and PK enzymes were used to assess the role of berberine hydrochloride in regulating glycolytic function of the intestine.

### Quantitative fluorescence RT-PCR

Total gut RNA was extracted using the Trizol (Invitrogen) method and subjected to cDNA synthesis. The RNA concentration was quantified by spectrophotometry (Eppendorf), while agarose gel electrophoresis was employed to assess RNA quality. Subsequently, 1 µg of RNA from each sample was used as a template for reverse transcription according to the instructions (TransGen, Beijing, China) to obtain cDNA. Quantitative fluorescence PCR was conducted on an ABI 7300 instrument using the Power SYBR Green PCR Master Mix kit, and the relative expression levels were calculated using the 2^−ΔΔCt^ method. Three samples were analyzed for each gene, and the average Ct value of three replicates from each sample was used to calculate the ΔCt relative to the internal reference gene. For tissue samples, 2^−ΔΔCt^ was used to calculate the relative expression of the target gene. TransStart Top Green qPCR SuperMix (TransGen) was used for RT-qPCR.

The primers used for RT-qPCR analysis were as follows: NFκB, Cu–Zn SOD, mMnSOD, ZO-1, ZEB1, occludin, STAT5b, and beta-actin (Table 1). The NFκB, ZO-1, ZEB1, occludin, and STAT5b genes were selected to assess the improvement of intestinal epithelial barrier function by berberine hydrochloride. The Cu–Zn SOD and mMnSOD genes, as well as the enzymes MDA, POD, and LPO, were used to assess the ability of berberine hydrochloride to cope with oxidative stress in the intestine.

**Table 1:** Primers used for the real-time PCR analysis of *Charybdis japonica* genes

Gene	Forward primer (5'- > 3')	Reverse primer (5'- > 3')	Annealing temperature (℃)	Size
				**(bp)**
mMnSOD	GTGTGTTCTCCAGCCCAATG	TAGTGACCGCCAGTGGATAC	60	183
Cu-ZnSOD	TTGCTGTTCAAGGTTCTGGC	AGTAAGCATGCTCCCAGACA	60	150
STAT5b	AGTGTCCTGGGATAATGCGT	GAAAGCCTTCCCTGCCAAAA	60	166
ZEB1	AAACACCTACCAGAGCTCCC	CACTGCTGGTCACATGCTTT	60	194
OCCLUDIN	TCAACGGAGCAGATACAA	CCAGGTCAGTGAAGGGTC	60	101
ZO1	CCTCAGGCTTAAAGTTCC	AAAGGTGGGATAGAAGGA	60	131
NFκB	CCATTGGGGTTCCTTCTCCT	TAGGGAAGGCAGCTCTGTTC	60	198
Beta -actin	CTGCGGAATCCACGAAAC	GTCAGCAATGCCAGGGTA	60	121

### Data analysis

All statistical analyses were performed using Microsoft Office Excel 2010 software and SPSS 19.0. Data are presented in the form of the mean ± standard error. The 2^−ΔΔCt^ method was employed to analyze the relative mRNA levels of the target genes. Analysis of variance (ANOVA) was used to assess the significance of the mean values, with a significance level set at *P* < 0.05. Additionally, Duncan's test was used to conduct multiple comparisons. In this experiment, we integrated previously published data from bioRxiv that pertained to the infection of C. japonica by A. hydrophila. This dataset included information on enzyme activity, CK levels, and AH data of 16 s gut microbes [26].

## Results

### *C. japonica* survival rate of *C. japonica* immersed in berberine hydrochloride infected by *A. hydrophila*

**Fig. 1:**
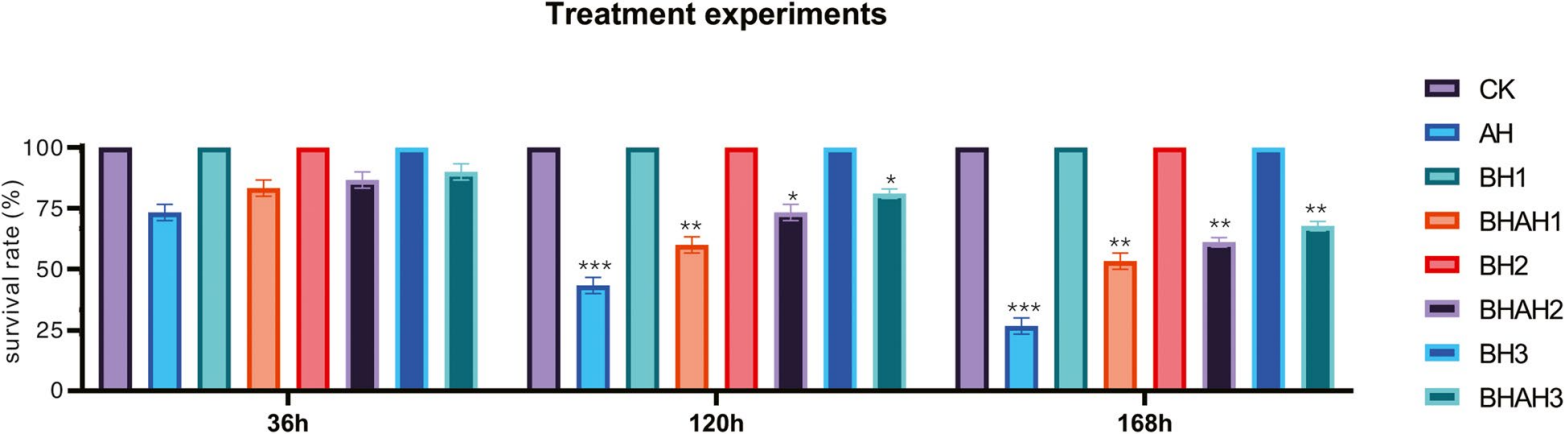
The survival rate of *C. japonica* in eight groups. *C. japonica* infected with 10^5^ CFU/L *A. hydrophila*, immersed in berberine hydrochloride at 100 (BH1), 200 (BH2), and 300 mg/L berberine hydrochloride (BH3), and crabs infected with 10^5^ CFU/L *A. hydrophila* and immersed in berberine hydrochloride at 100 (BHAH1), 200 (BHAH2), and 300 mg/L (BHAH3), and the control group CK

*C. Japonica* infected with *A. hydrophila* at 168 h had the lowest survival rate. The survival rate of *C. japonica* infected with *A. hydrophila* and soaked with 300 mg / L of berberine hydrochloride was 73% (Fig. 1). As the concentration increased, the survival rate gradually increased and the mortality rate decreased.

### Annotation and evaluation of microbial species in the gut

**Fig. 2:**
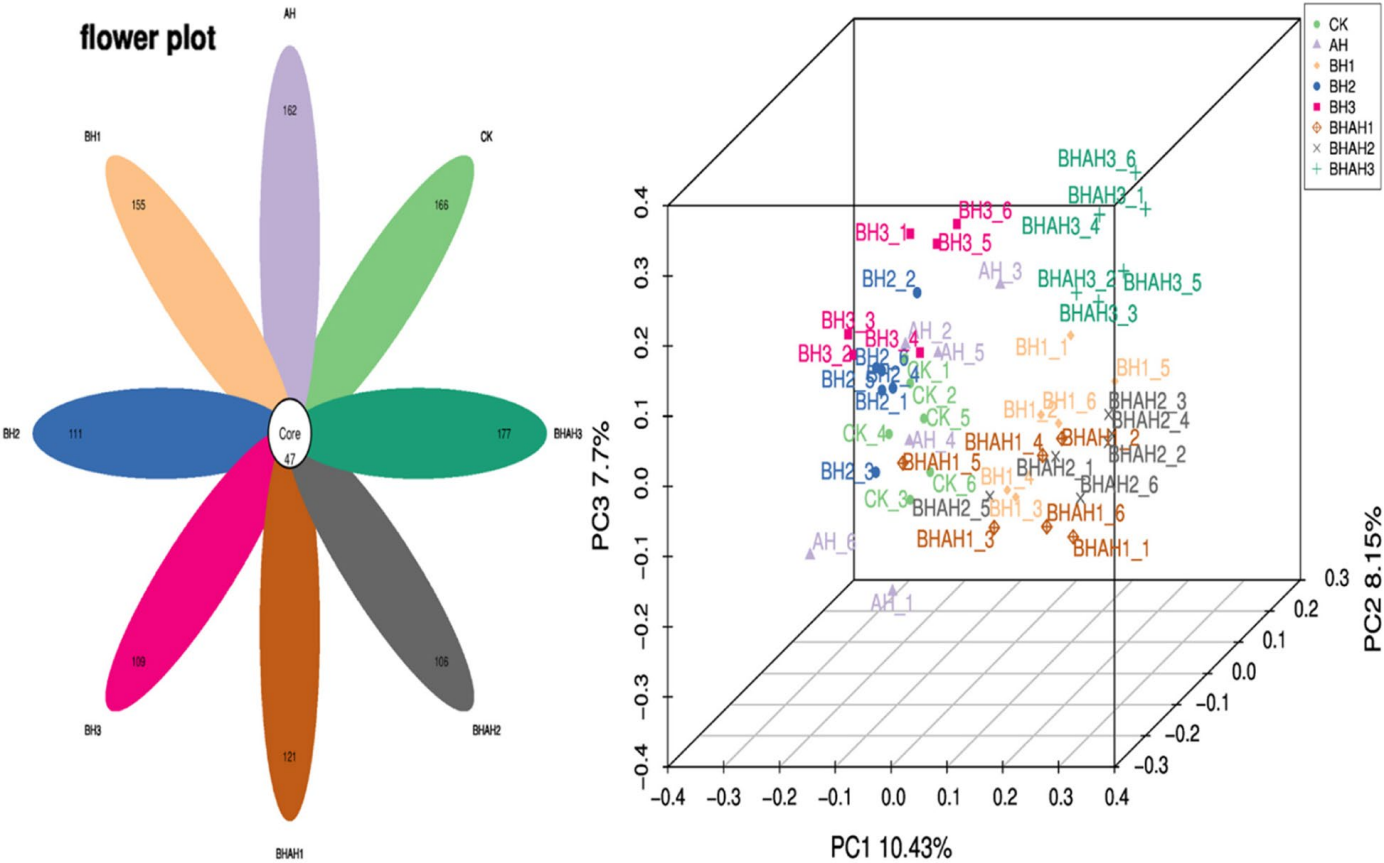
Distribution of ASVs Petal diagram and PCoA (3D), the number in Core in the left figure represents the ASVs shared by all samples (Core ASVs), and the number on the petal represents the total ASVs of each sample minus the number of common ASVs. Right figure: The same color means the same grouping. A point is a sample, and similar samples will gather together. *C. japonica* infected with 10^5^ CFU/L *A. hydrophila*, crabs immersed in berberine hydrochloride at 100 (BH1), 200 (BH2), and 300 mg/L berberine hydrochloride (BH3), and crabs infected with 10^5^ CFU/L *A. hydrophila* and immersed in berberine hydrochloride at 100 (BHAH1), 200 (BHAH2), and 300 mg/L (BHAH3), and the control group CK

After removing low quality reads, a total of 2174,620 valid reads and 1,273,965 ASV were obtained from 48 samples by high-throughput sequencing (Fig. 2 and Table 2). Each point in the PCA(3D) analysis graph represents a sample with the same color for the same grouping. Samples with the same color were clustered together and had some distance from samples with different staining, proving that there were some differences between groups. There were 47 ASV common to *C. japonica* in the eight groups. There were 166 ASV specific to CK and 162 ASV specific to *A. hydrophila* infection. In addition, there were 155 ASV specific to BH1, 111 ASV specific to BH2, 109 ASV specific to BH3, 121 ASV specific to BHAH1, 106 ASVs specific to BHAH2, and 177 ASV specific to BHAH3.

**Table 2:** Statistical table of the process of dada2 denoising to generate ASVs

sample-id	Raw_reads	filtered	Percentage of input passed filter	denoised	merged	ASV_counts	Total_ASVs
AH_1	51,820	50,003	96.49	49,705	49,285	42	618
AH_2	53,801	51,465	95.66	50,883	48,316	116	618
AH_3	40,419	39,217	97.03	38,513	36,273	98	618
AH_4	48,431	46,241	95.48	45,908	44,464	87	618
AH_5	58,952	56,650	96.1	56,113	53,284	113	618
AH_6	49,210	47,255	96.03	47,078	46,507	43	618
BH1_1	49,286	47,591	96.56	47,104	44,883	119	618
BH1_2	42,317	40,544	95.81	40,308	39,888	58	618
BH1_3	56,653	53,787	94.94	53,430	52,157	76	618
BH1_4	38,914	37,165	95.51	36,826	35,702	76	618
BH1_5	45,133	43,432	96.23	43,025	42,018	71	618
BH1_6	33,917	32,662	96.3	32,416	31,353	72	618
BH2_1	41,506	39,792	95.87	39,520	38,878	72	618
BH2_2	46,436	44,293	95.39	43,860	42,423	90	618
BH2_3	35,174	33,759	95.98	33,549	32,986	69	618
BH2_4	30,976	29,392	94.89	29,193	28,812	49	618
BH2_5	38,046	36,328	95.48	36,070	35,307	64	618
BH2_6	46,160	43,871	95.04	43,560	42,829	61	618
BH3_1	37,223	35,638	95.74	35,216	33,644	84	618
BH3_2	47,972	45,815	95.5	45,528	44,553	71	618
BH3_3	36,683	34,497	94.04	34,164	33,637	67	618
BH3_4	37,787	35,866	94.92	35,665	34,701	64	618
BH3_5	46,698	44,572	95.45	44,157	43,153	73	618
BH3_6	46,961	45,024	95.88	44,784	43,831	85	618
BHAH1_1	38,472	37,190	96.67	36,855	36,189	56	618
BHAH1_2	40,394	38,743	95.91	38,409	37,784	72	618
BHAH1_3	50,894	49,038	96.35	48,506	46,679	75	618
BHAH1_4	49,105	47,093	95.9	46,666	45,304	80	618
BHAH1_5	45,512	43,768	96.17	43,517	42,812	62	618
BHAH1_6	52,286	50,419	96.43	49,819	48,418	63	618
BHAH2_1	37,361	35,375	94.68	34,979	34,070	55	618
BHAH2_2	42,003	39,918	95.04	39,556	38,284	60	618
BHAH2_3	41,031	39,642	96.61	39,243	38,302	78	618
BHAH2_4	53,817	51,245	95.22	50,705	48,603	94	618
BHAH2_5	41,253	39,287	95.23	38,989	37,895	61	618
BHAH2_6	42,252	40,627	96.15	40,267	39,003	72	618
BHAH3_1	44,334	42,778	96.49	42,416	40,773	115	618
BHAH3_2	51,639	49,524	95.9	49,091	46,732	104	618
BHAH3_3	55,497	53,244	95.94	52,973	52,339	70	618
BHAH3_4	50,830	48,608	95.63	48,129	46,543	110	618
BHAH3_5	60,984	58,427	95.81	58,070	57,119	87	618
BHAH3_6	41,347	39,546	95.64	39,185	37,927	111	618
CK_1	52,331	50,441	96.39	49,783	47,004	109	618
CK_2	48,898	47,062	96.25	46,525	44,069	105	618
CK_3	38,808	37,177	95.8	36,879	36,048	81	618
CK_4	43,823	42,159	96.2	41,898	41,466	54	618
CK_5	58,821	56,848	96.65	56,183	53,737	108	618
CK_6	32,453	30,809	94.93	30,281	28,774	78	618

**Fig. 3:**
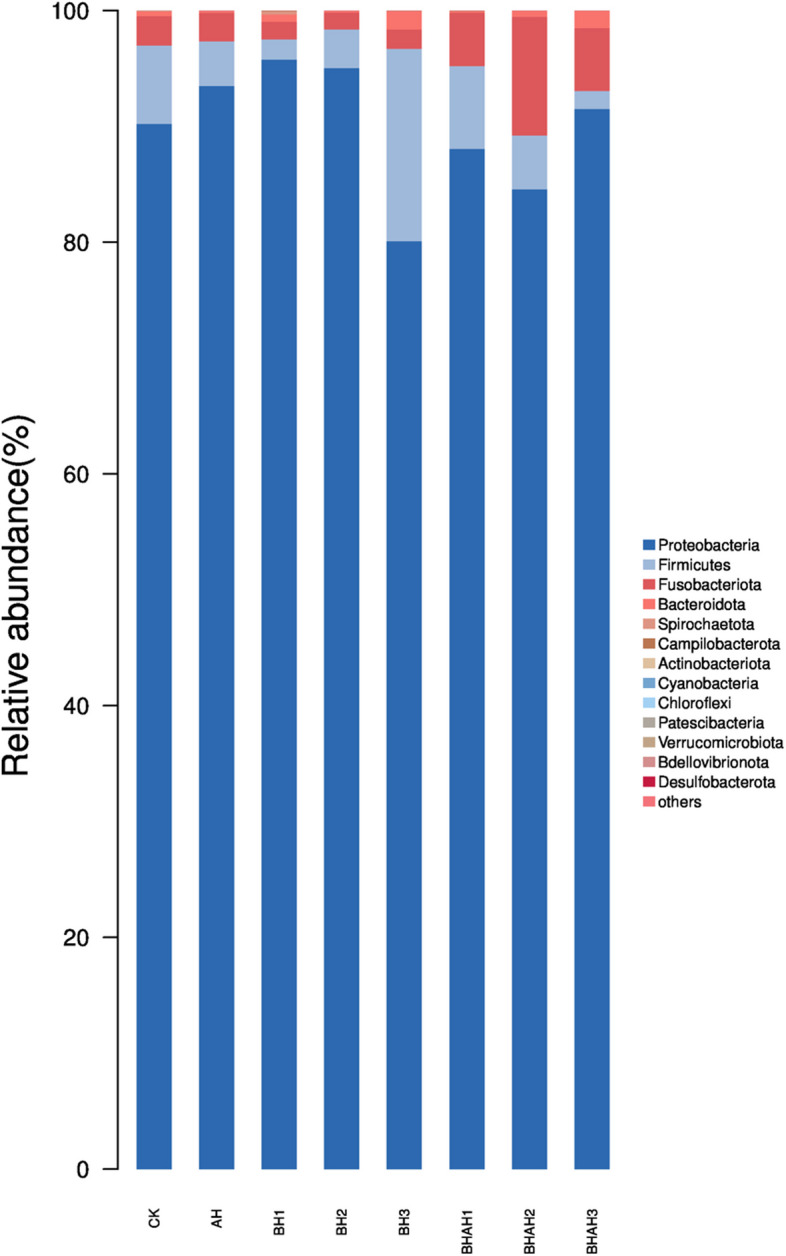
A histogram depicting the relative distribution of each group at the phylum level, specifically focusing on the top 15 species with the highest relative abundance. The sequence number percentage represents the ratio of the number of annotated sequences at the phylum level to the total annotated dataset. The colors used in the histogram correspond to the color sequence provided in the legend located on the right side

**Fig. 4:**
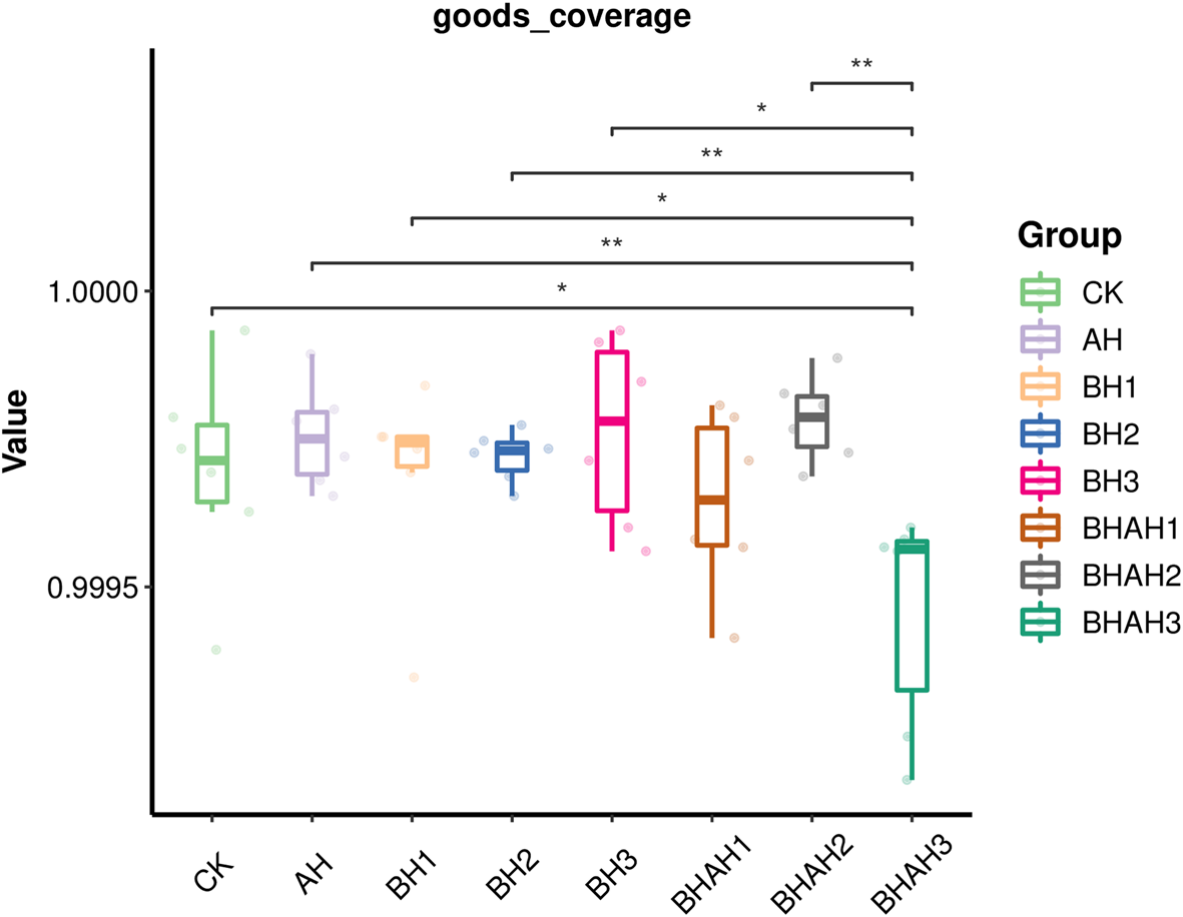
Boxplot for inter-group comparison of ASV diversity index in eight groups (AH, BH1, BH2, BH3, BHAH1, BHAH2, BHAH3 and CK)

The abundance of intestinal microorganisms could be divided into phylum, class, order, family, and genus. In the 48 samples from the groups, 99% of the system types belonged to only four core phyla: Proteobacteria (90%), Firmicutes (5.35%), Fusobacteria (3.54%), and Bacteroidetes (0.71%; Fig. 3). Under different concentrations of berberine hydrochloride and after *A. hydrophila* infection, the distribution of the four dominant phyla in each sample was similar, but the abundance and variation trends were different. In the analysis of Good’s coverage diversity (Fig. 4), the main two factors in the 48 samples were the richness and evenness of species composition. There were obvious differences in the Shannon index of the species detected in the AH, BH1, BH3, BHAH1 BHAH2, BHAH3 and CK groups (Fig. 4).

### The Impact of Potential Metabolic Pathways in the intestinal flora

**Fig. 5:**
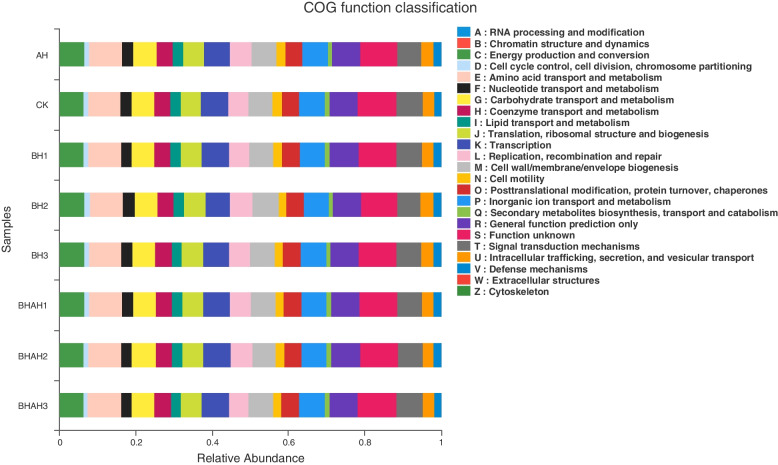
Histogram of PICRUSt functional classification statistics differences among the groups (AH, BH1, BH2, BH3, BHAH1, BHAH2, BHAH3 and CK)

We investigated the effect of berberine hydrochloride on the metabolic pathways in the intestinal flora of *C. japonica* using PICRUSt analysis methods. Figure 5 shows that intestinal flora metabolism was enhanced in the berberine hydrochloride treatment group. For example, energy production and conversion, Carbohydrate transport and metabolism, and Lipid transport and metabolism of the intestinal flora when infected with *C. japonica* immersed in a concentration of 300 mg/L berberine hydrochloride.

### Phylogenetic analysis of the species genus

**Fig. 6:**
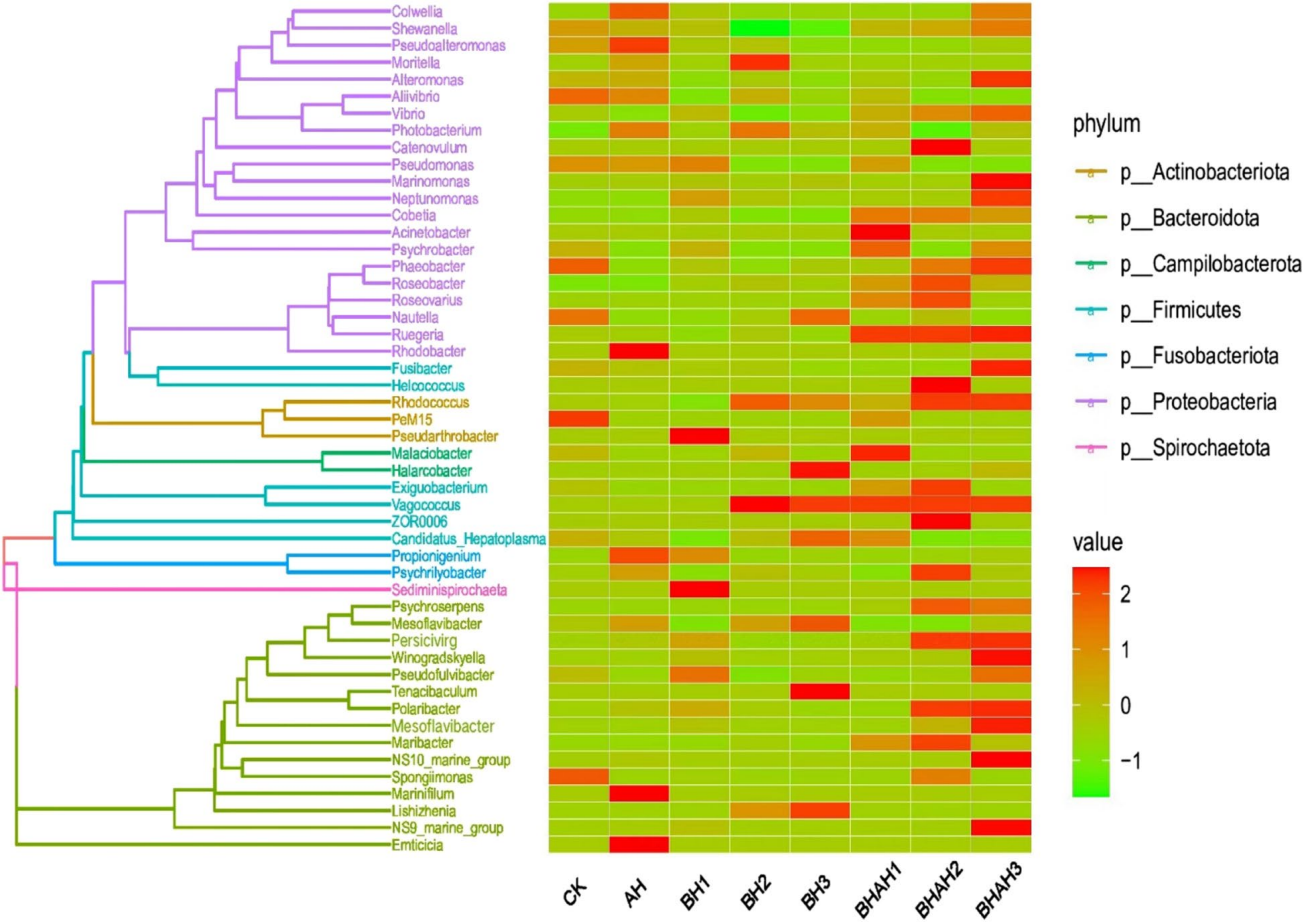
Phylogenetic evolutionary tree and the heat map of abundance distribution between different groups. The left is the evolutionary tree, with different populations are distinguished by different colors. The closer the branches of the evolutionary tree, the closer the kinship and branches at each end representing an ASV. The genus classification to which the ASVs belong was annotated. The right is the standardized abundance: The absolute abundance of each sample minus the mean absolute abundance of the species divided by the standard deviation gives a standardized mean abundance of -1and standard deviation of 2. 144 samples were divided into eight groups: AH, BH1, BH2, BH3, BHAH1, BHAH2, BHAH3, and CK

In this study, 200 and 300 mg/L of berberine hydrochloride could effectively increase the relative abundance of *Phaeobacter ssp*., *Ruegeria ssp*., *Cobetia ssp.*, *Exiguobacterium ssp*., *Vagococcus ssp*. and *Rhodococcus ssp*. (Fig. 6). Berberine hydrochloride concentrations of 300 mg/L were found to effectively increase the relative abundance of *Polaribacter ssp*., *Persicivirg ssp*. and Mesoflavibacter ssp.

### Analysis of enzyme activities and genes

**Fig. 7:**
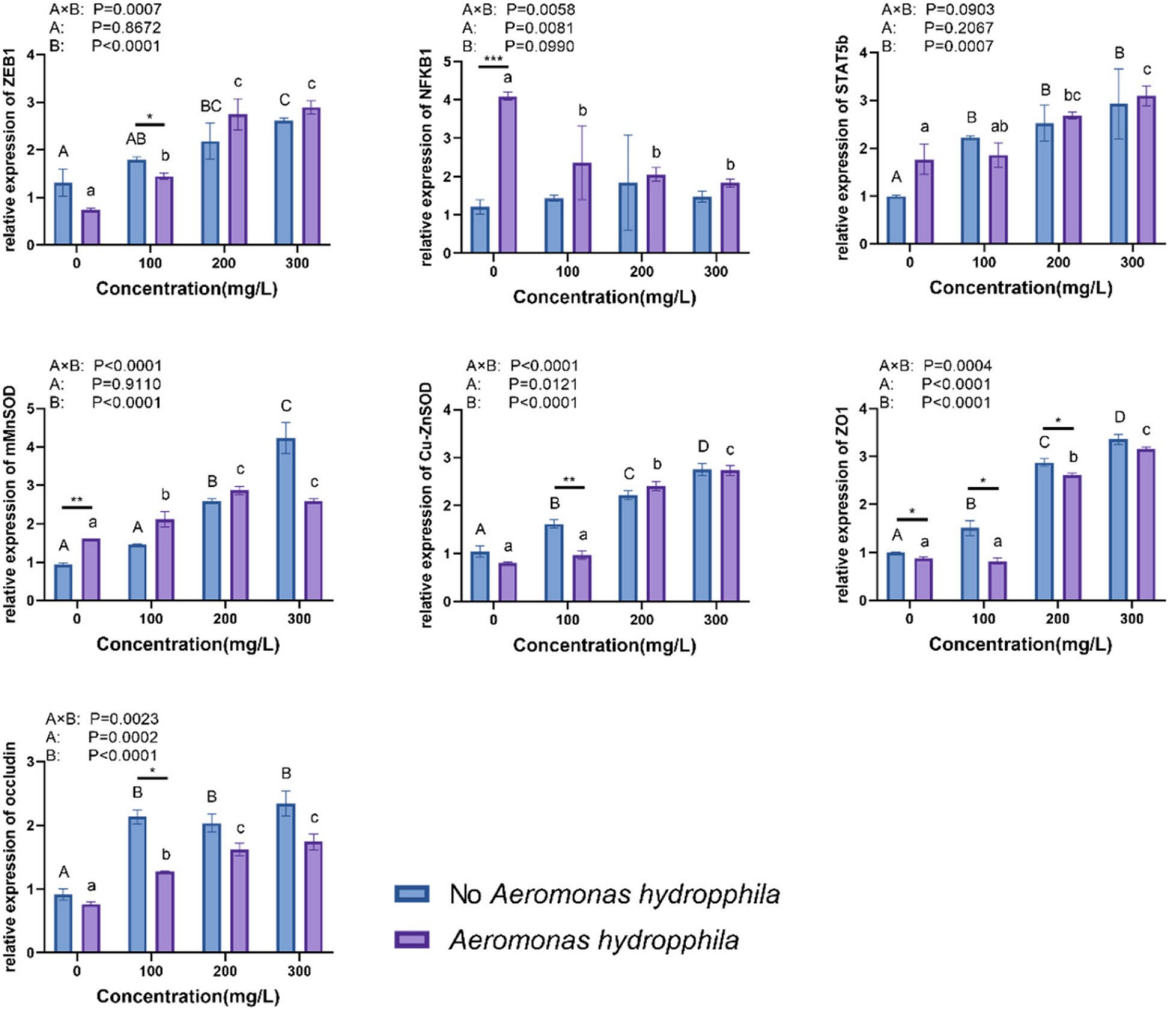
The mRNA expression levels of genes associated with the immune response were analyzed in the liver, gut, and pancreas of *C. japonica*. The experimental groups of *C. japonica* were exposed to different concentrations of berberine hydrochloride (0 mg/L, 100 mg/L, 200 mg/L, and 300 mg/L) either alone or in combination with an injection of *A. hydrophila* at a concentration of 10^5 CFU/L. The results are presented as the mean ± standard deviation (SD) with three replicates (*n* = 3). lowercases were used for the significant differences of groups injected with *A. hydrophila*, and the significant differences of groups without *A. hydrophila* injection were presented with capital letter. *and ** significant difference (*p* < 0.05) were used for the significant differences of *C. japonica* under *A. hydrophila* challenge or not, which immersed in same concentration of berberine hydrochloride. Lowercase letters indicate significant differences in the *A. hydrophila* group. Capital letters indicate significant differences in the No *A. hydrophila* group

**Fig. 8:**
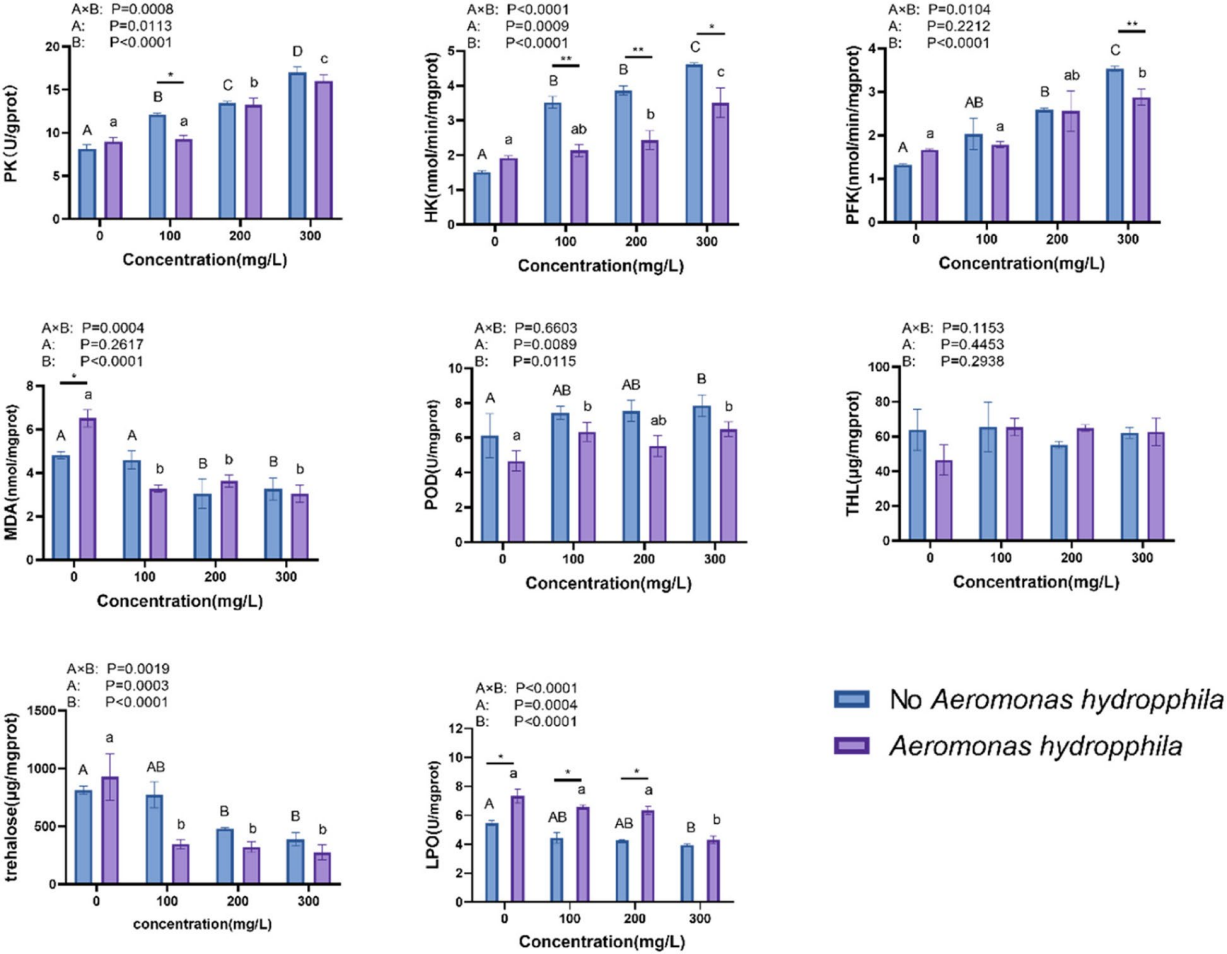
The intestinal related enzyme activities and contents of the *C. japonica* intestine were determined, and crabs were immersed in 0, 100, 200, and 300 mg/L of berberine hydrochloride alone or injected with *A hydrophila* at 10^5^ CFU/L. Values are given as mean ± SD (*n* = 3). lowercases were used for the significant differences of groups which injected with *A. hydrophila*, and the significant differences of groups without *A. hydrophila* injection were presented with capital letter. *and ** significant difference (*p* < 0.05) were used for the significant differences of *C. japonica* under *A. hydrophila* challenge or not, which immersed in same concentration of berberine hydrochloride. Lowercase letters indicate significant differences in the *A. hydrophila* group. Capital letters indicate significant differences in the No *A. hydrophila* group

Figure 7 shows that *C. japonica* significantly increased the activities of PK, PFK, HK, and THL in the intestinal. PK, PFK, HK, and THL in intestinal tract increased significantly and to their maximum with berberine hydrochloride concentrations of 300 mg/L. Supplementation of berberine hydrochloride at doses of 100, 200, and 300 mg/L could reduce the level of trehalose. The addition of berberine hydrochloride increased intestinal POD activity and decreased LPO and MDA content compared to the control group. The primers for each gene used in this analysis are shown in Table 1 and Fig. 8. *C. Japonica* treated with 200 and 300 mg / L of berberine hydrochloride with or without *A. hydrophila* injection significantly increased the levels of Cu–Zn SOD, MnSOD, ZO-1, ZEB1, occludin, and STAT5b expression in the intestinal tract. The levels of Cu–Zn SOD, MnSOD, ZO-1, ZEB1, occludin and STAT5b expression in the intestinal tract were significantly increased when *C. japonica* was supplemented with berberine hydrochloride at 200, and 300 mg/L. NFκB gene expression was down-regulated in *C. japonica* soaked in berberine hydrochloride.

## Discussion

The intestinal microbiota plays a crucial role in the gastrointestinal tract and greatly impacts the well-being of the host organism. When microbial dysbiosis occurs, it can have harmful effects on the gastrointestinal tract, leading to inflammation and changes in the permeability of the intestinal epithelium. In this context, berberine hydrochloride has shown both antibacterial and antioxidant properties in various in vitro and in *vivo studies* [27]. This compound leverages its alkaloid properties to indirectly disrupt the bioavailability of the intestinal flora, thereby modulating the composition of intestinal microbes [27]. In the treatment of *C. japonica* infection with *A. hydrophila*, berberine hydrochloride can modulate LZM enzyme activity to remove harmful bacteria [28]. The highest survival rate of *C. japonica* infected with *A. hydrophila* was found in this study following treatment with 300 mg/L berberine hydrochloride.

Probiotics can prevent pathogens from proliferating in the gut and stimulate the host's immune system to improve the health status of crustaceans [29]. Thus, probiotics can protect host health by regulating the intestinal flora [2, 30, 31]. Studies have found that *Vagococcus fluvialis* has an effect on the cellular immune system of European sea bass (*Dicentrarchus labrax)* and that *Vagococcus fluvialis* has elevated phagocytic activity in macrophages of sea bass incubated with UV-inactivated bacteria [32]. *Exiguobacterium acetylicum* regulates the expression of cytokine genes in Carfish to protect against infection by *A. hydrophila* and has some resistance to disease [33]. Some related bacteria reported in the genera *Phaeobacter*, *Ruegeria*, *Cobetia* and *Rhodococcus* were found to prevent Vibrio from proliferating in the intestinal track [34–37]. In the present study, 200 and 300 mg / L of berberine hydrochloride were found to be effective at increasing the relative abundance of bacteria from the genera *Phaeobacter*, *Ruegeria*, *Cobetia, Exiguobacterium*, *Vagococcus* and *Rhodococcus*. From the above conclusions, it can be seen that an increase in the abundance of these probiotics can suppress pathogenic bacteria through a quantitative advantage.

In addition, berberine hydrochloride was found to increase the relative abundance of algal polysaccharide-degrading probiotic bacteria in this study. Some of the bacteria of the genera *Polaribacter*, *Persicivirga* and *Mesoflavibacter* can break down polysaccharides. Studies over the years have found that *Polaribacter staleyi* can degrade algal polysaccharides [38], while *Persicivirga ulvanivorans* is a probiotic strain capable of degrading green algal polysaccharides [38]. *Mesoflavibacter zeaxanthinifaciens* is a xylanase producing probiotic [39]. The present study found that soaked in 200 and 300 mg/L berberine hydrochloride before *A. hydrophila* infection with *C. japonica* was effective in increasing the relative abundance of the genus *Polaribacter* and *Persicivirga*. Concentrations of 200 and 300 mg/L berberine hydrochloride were effective in increasing the relative abundance of the genus *Mesoflavibacter*. Alginose content decreased with increasing concentrations of berberine hydrochloride. However, *C. japonica* soaked in 300 mg/L berberine hydrochloride in the intestinal flora of energy production and conversion, carbohydrate transport and metabolism, and lipid transport and metabolism were very high. From the above findings, it is clear that berberine hydrochloride can help the genera *Polaribacter, Persicivirg* and *Mesoflavibacter* break down polysaccharides more efficiently, and that at high concentrations, berberine hydrochloride can increase the energy metabolism of the intestinal flora of *C. japonica* [34]. Berberine hydrochloride also activates THL to reduce algal sugars. These processes can release energy.

Berberine hydrochloride has a strong role in regulating the glycolytic process in aquatic organisms [28]. Glycolysis is a metabolic pathway that involves the enzymatic breakdown of glucose into pyruvate, resulting in the generation of adenosine triphosphate (ATP). Hepatic kinase (HK) facilitates the process of phosphorylating glucose, resulting in the production of glucose-6-phosphate. This phosphorylation event serves as the initial step in the glycolytic pathway [40]. The terminal position of the glycolytic pathway is occupied by pyruvate kinase (PK). Pyruvate kinase (PK) serves as the rate-limiting enzyme in the glycolysis pathway, facilitating the transfer of phosphate groups from phosphoenolpyruvate to adenosine diphosphate (ADP). This enzymatic reaction results in the production of pyruvate and adenosine triphosphate (ATP) [41]. In one study, it was found that treatment of hepatocytes with 5, 20, and 50 μmol/ L of berberine hydrochloride increased glucose consumption, enhanced gluconeogenesis, and regulated the mRNA levels of hexokinase [42]. These results suggest that *C. japonica* infected with *A. hydrophila* showed an increase in the activity of the HK, PFK and PK enzymes with increasing concentrations of berberine hydrochloride. This phenomenon revealed that berberine hydrochloride could repair organismal damage by regulating the energy released in the gut of *C. japonica* infected with *A. hydrophila*.

Berberine hydrochloride can improve the damaged intestinal epithelial barrier [43]. The intestinal epithelium consists of intestinal epithelial cells and tight junction proteins that maintain intramucosal homeostasis. Berberine hydrochloride upregulates the mRNA levels of proteins associated with intestinal tight junction proteins (ZO-1, ZEB1 and occludin) to ameliorate damage to the intestinal epidermal barrier [43]. Components of the Stat5b activation pathway play an unexpected role in epithelial cell homeostasis and response to injury [44]. STAT5b may also directly inhibit NFκB signalling, and NFκB activity is enhanced following the knockdown of STAT5b. NFκB plays a predominantly pro-inflammatory role in the gut [44]. In other animal studies, berberine hydrochloride reduced NFκB activity in the intestine [43]. In the present study, ZO-1, ZEB1, occludin and STAT5b genes were up-regulated and NFκB genes were down-regulated in *C. japonica* soaked in berberine hydrochloride. It can be speculated that berberine hydrochloride improves intestinal barrier function by increasing the expression of ZO-1, ZEB1, occludin and STAT5b in *C. japonica*.

The positive effect of berberine hydrochloride on immunity may be associated with a reduction in oxidative damage in *C. japonica* exposed to optimal levels of berberine hydrochloride. Berberine hydrochloride protects cells by altering the activity of endogenous antioxidants to inhibit apoptosis [45]. As an important antioxidant enzyme, SOD is responsible for catalyzing the conversion of O_2-_ to O_2_ and H_2_O_2 _[46, 49]_._ H_2_O_2_ is converted to H_2_O by the CAT enzyme, and when antioxidant enzyme is activated, there is less disruption of the cell membrane and low levels of MDA are released. In this study, Cu–Zn SOD and MnSOD gene expression was higher in *C. japonica* liver. The addition of berberine hydrochloride increased intestinal POD activity and decreased LPO and MDA content compared to the control group, thereby increasing the hepatic antioxidant capacity and scavenging free radicals in *C. japonica*.

## Conclusion

In summary, Illumina sequencing technology was used to analyze the different physiological states of *C. japonica* during exposure to berberine hydrochloride. Intestinal POD activity and reduced LPO and MDA levels were found. The addition of berberine hydrochloride increased the amount of energy released during carbohydrate metabolism *C. japonica*. Berberine hydrochloride improved intestinal barrier function by increasing the expression of ZO-1, ZEB1, occludin and STAT5b in *C. japonica*. This study adds to our understanding of the response of crustacean gut microbes to berberine hydrochloride.

### Supplementary Information

Supplementary Material 1.

## Data Availability

The datasets presented in this study can be found in online repositories. The names of the repository/repositories and accession number(s) can be found below: https://www.ncbi.nlm.nih.gov/, SRA: SRP358796, Bioproject accession: PRJNA804319.
