# Supplementary material for: Effects of berberine hydrochloride on antioxidant response and gut microflora in the Charybdis japonica infected with Aeromonas hydrophila
Source: BMC Microbiol. 2024 Aug 2;24:287. doi: 10.1186/s12866-024-03420-3 (PMC11295712; doi:10.1186/s12866-024-03420-3)
Supplement: Supplementary file 1 — Supplementary Material 1. [file 12866_2024_3420_MOESM1_ESM.docx]

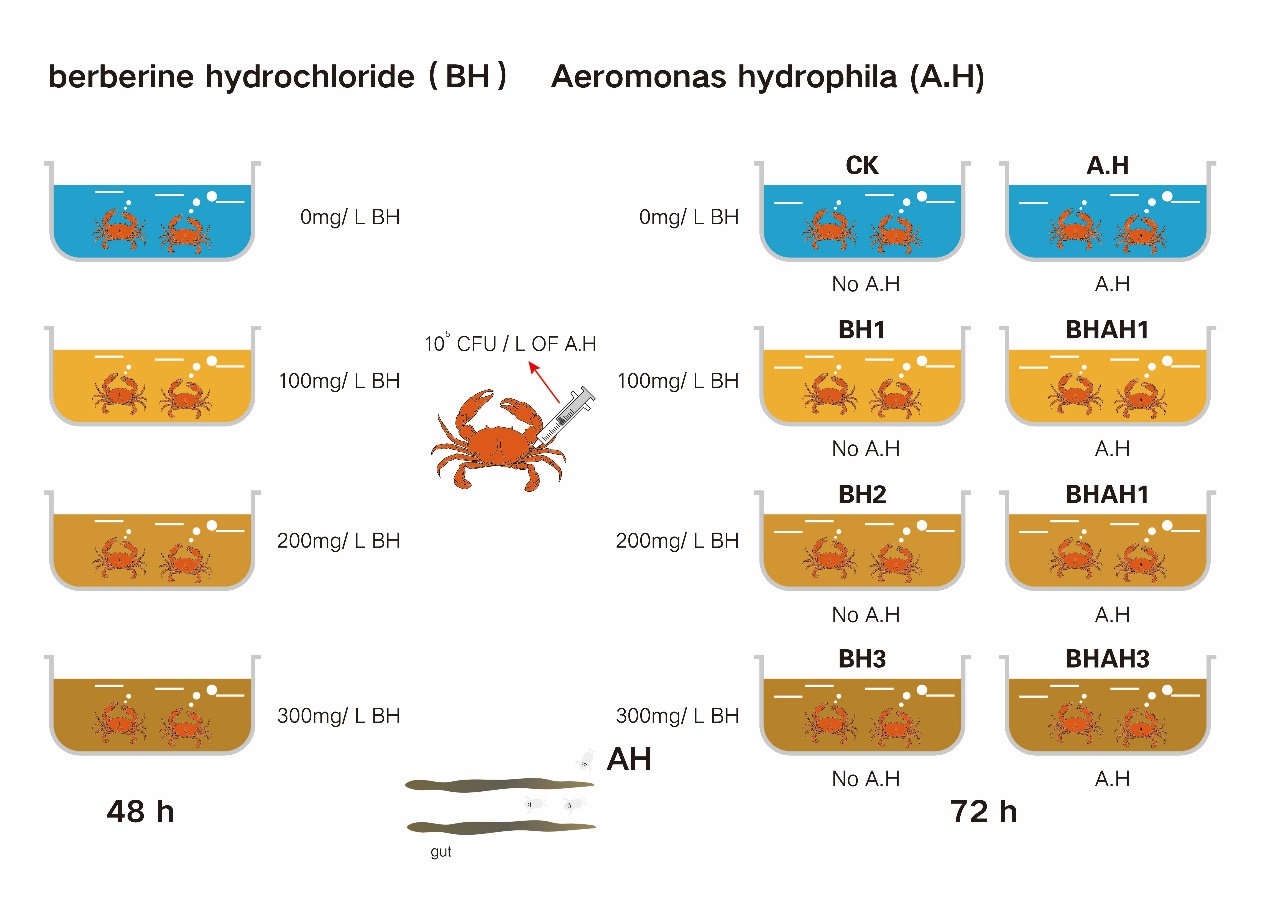


Figure S1. Experimental flow chart. 48 samples were divided into eight groups: *C. japonica* infected with 10^5^ CFU/L *A. hydrophila* and immersed in berberine hydrochloride at 100 (BH1), 200 (BH2), and 300 mg/L (BH3); crabs infected with 10^5^ CFU/L *A. hydrophila* and immersed in berberine hydrochloride at 100 (BHAH1), 200 (BHAH2), and 300 mg/L (BHAH3), and the control group CK.

Figure S2. *C.J* (720) were divided into eight groups, infected with *A.H*, *C. J* soaked in berberine hydrochloride: *C.J* injected with 10^5^ *A.H* and soaked in berberine hydrochloride: the proportions of BHAH1 were 100 mg/L, the proportions of BHAH2 were 200 mg/L, the proportions of BHAH3 were 300 mg/L, and the control group CK. The sample survival numbers were checked at 50, 100,150 and 200 h.
